# Estimating the mean and variance from the median, range, and the size of a sample

**DOI:** 10.1186/1471-2288-5-13

**Published:** 2005-04-20

**Authors:** Stela Pudar Hozo, Benjamin Djulbegovic, Iztok Hozo

**Affiliations:** 1 Indiana University Northwest, Department of Mathematics, Gary, IN, 46408 USA; 2 Interdisciplinary Oncology Program, H. Lee Moffitt Cancer Center and Research Institute at the University of South Florida, Tampa, FL, USA

## Abstract

**Background:**

Usually the researchers performing meta-analysis of continuous outcomes from clinical trials need their mean value and the variance (or standard deviation) in order to pool data. However, sometimes the published reports of clinical trials only report the median, range and the size of the trial.

**Methods:**

In this article we use simple and elementary inequalities and approximations in order to estimate the mean and the variance for such trials. Our estimation is distribution-free, i.e., it makes no assumption on the distribution of the underlying data.

**Results:**

We found two simple formulas that estimate the mean using the values of the median (*m*), low and high end of the range (*a *and *b*, respectively), and *n *(the sample size). Using simulations, we show that median can be used to estimate mean when the sample size is larger than 25. For smaller samples our new formula, devised in this paper, should be used. We also estimated the variance of an unknown sample using the median, low and high end of the range, and the sample size. Our estimate is performing as the best estimate in our simulations for very small samples (*n *≤ 15). For moderately sized samples (15 <*n *≤ 70), our simulations show that the formula range/4 is the best estimator for the standard deviation (variance). For large samples (*n *> 70), the formula range/6 gives the best estimator for the standard deviation (variance).

We also include an illustrative example of the potential value of our method using reports from the Cochrane review on the role of erythropoietin in anemia due to malignancy.

**Conclusion:**

Using these formulas, we hope to help meta-analysts use clinical trials in their analysis even when not all of the information is available and/or reported.

## Background

To perform meta-analysis of continuous data, the meta-analysts need the mean value and the variance (or standard deviation) in order to pool data. However, sometimes, the published reports of clinical trials only report the median, range and the size of the trial. In this article we use simple and elementary inequalities in order to estimate the mean and the variance for such trials. Our estimation is distribution-free, i.e., it makes no assumption on the distribution of the underlying data. In fact, the value of our approximation(s) is in giving a method for estimating the mean and the variance exactly when there is no indication of the underlying distribution of the data. In current practice, the median is often substituted for the mean, and the Range/4 or Range/6 for the standard deviation. However, it has not been shown that median can indeed be used to replace mean values, nor when the range-formulas are appropriate.

## Methods

### Assumptions

Suppose a clinical trial reports the following summary measures for a certain event:

*m *= Median

*a *= The smallest value (minimum)

*b *= The largest value (maximum)

*n *= The size of the sample.

In this article, we want to estimate the mean, and the standard deviation of this sample of size *n*. First we will order this sample by size:

*a *= *x*_1 _≤ *x*_2 _≤ *x*_3 _≤ … *x*_*M*-1 _≤ *x_M _*= *m *≤ *x*_*M*+1 _≤ … ≤ *x*_*n*-1 _≤ *x*_*n *_= *b*,

where the *M*^th ^number is the median, and  (for the sake of simplicity, we will assume that *n *is an odd number).

## Results

### Estimating the sample mean 

We begin with several simple inequalities:



Adding up and diving by *n*, the middle column is exactly the sample mean, .

Adding up and diving by *n *for all three columns, we get the following inequality:



After replacing  into the inequality above, we get:



Therefore, the lower bound for the sample mean is



The upper bound for the sample mean is



The sample mean can than be estimated as

, or



When the size of the sample is fairly large, the second fraction becomes negligible and the estimate can be written in a simplified form:



We can use this simple expression even if we do not know the size of the sample. The length of the interval which contains the sample mean (the interval [LB, UB]), is approximately



### Estimating the sample variance

Even when the only information we have about a set of data is it's range: *R *= *b *- *a*, we can still estimate the standard deviation. If our data are normally distributed, then *P*[-2*σ *<*X *- *μ *< 2*σ*] = 0.95, and therefore, the range covers approximately 4*σ*, i.e., .

When the data we are dealing with are not normally distributed, we can still use the Chebyshev's inequality [1,2], and obtain the following for *k *= 3: . Therefore, the range covers approximately 6*σ*, i.e., .

On the other hand, if the summary results for a clinical trial include the median and the size of the sample, we can presumably do better than the two range approximations above. Next section deals with that situation.

### The Variance S^2 ^– distribution free inequalities

Using the inequalities (1) and taking in consideration that all the data are non-negative, we can multiply each row *i *with the value *x*_i _(*i *= 1, 2, 3, ..., *n*). We obtain the following inequalities:



Adding up by columns, we have the following:



Using the inequalities (1) again, we estimate the sums in LB and UB as



Therefore, the expressions in (7) can be estimated as



The sum of squares can be therefore estimated as



The sample variance can be evaluated from the computational formula



We can estimate  using (10) and  using (4). Therefore, after simplifying:



Note that if we let *n *grow without bound, the expression (12) becomes the well-known range formula .

### The Variance S^2 ^– equidistantly spaced data

The formula (4) can also be obtained by dividing the range [*a*, *b*] into two parts: [*a*, *m*), and [*m*, *b*]. We then subdivide each of these two parts into subintervals using equally spaced partition points. In other words, we are estimating each of the data points (except for *a*, *m*, and *b*) with uniformly spaced approximate points:



and



Therefore our sample is approximately given as

*a *= *x*_1 _≤ *x*_2 _≤ *x*_3 _≤ … ≤ *x*_*M*-1 _≤ *y*_1 _= *m *≤ *y*_2 _≤ … ≤ *y*_*M*-1 _≤ *y*_*M *_= *b*.

We can use this partition to estimate the sample variance  (and standard deviation, *S*). After a little algebra, the sample variance can be estimated by



If we let the number of estimation points increase without bounds, i.e., assume that *n *in the expression (15) is very large, we obtain a simplified version of the expression above:



## Discussion

### Analysis and performance of estimates

In order to verify the accuracy of these estimates, we ran several simulations using the computer package Maple where the data were variously distributed, and obtained the tables below.

We drew samples from five different distributions, Normal, Log-normal, Beta, Exponential and Weibull. The size of the sample ranged from 8 to about 100. In the first subsection we present the results of our estimation for a normal distribution, which is what meta-analysts would commonly assume. We also show the results of simulations where the data were selected from a skewed distributions. In each case we compared the relative error made by estimating the sample mean with the approximation given by formulas (4) and (5), as well as by the median, and the relative error made by estimating the sample variance by the formulas (12) and (16), as well as the well-known standard deviation estimators Range/4 and Range/6.

### Normal distribution

We drew 200 random samples of sizes ranging from 8 to 100 from a Normal Distribution with a population mean 50 and standard deviation 17. Then we graphed the average relative error vs. the sample size. Both estimators for the mean, formulas (4) and (5), are very close to the sample mean (within 4%). For sample sizes smaller than 29, formula (5) is actually outperforming the median as a mean estimator. For larger sample sizes, however, the median is more consistent estimator for a normally distributed sample.

The variance estimators however show greater distinction. For a very small sample size (up to 15) the formula (16) is performing the best (within 10% of the real sample standard deviation). When the sample size is between 16 and 70, the formula Range/4 is the best estimator of the sample standard deviation, with a relative error between 10–15%. However, for larger sample sizes, the formula Range/6 performs the best for this distribution. To compare the precision of these estimates on average, we collected the results of our simulation in the Additional file 1.

### Simulation with a skewed distribution (Log-Normal, Beta, Exponential and Weibull)

We also decided to run a simulation where the algorithm selects a sample from a skewed distribution. We decided to use Log-Normal distribution with parameters *μ *= 4, and *σ *= 0.3, Beta distribution with parameters *a *= 9 and *b *= 4, Exponential distribution with the parameter *λ *= 10 and Weibull distribution *a *= 2 and *b *= 35. These parameters were chosen arbitrarily, and the simulation results did not differ when we used different parameters (naturally, larger variance translates into larger relative error for mean estimators for any distribution). Just like in the case of Normal distribution, we ran our algorithm 200 times for each sample size ranging from 8 to 100. For each of the estimation formulas we then calculated the average relative error. We will summarize the best formula for estimation in Table 1.

**Table 1:** The best formula for estimation by distribution.

**Best Formula **for Sample size (n)	Mean Estimation		Standard Deviation Estimation		
	
	Formula (5)	Median	Formula (16)	Range/4	Range/6
Log-Normal	*n *≤ 23	23 <*n*	*n *≤ 15	15 <*n *≤ 64	64 <*n*
Beta	*n *≤ 30	30 <*n*	*n *≤ 15	15 <*n *≤ 100	100 <*n*
Exponential	*n *≤ 21	21 <*n*	*n *≤ 15	15 <*n *≤ 66	66 <*n*
Weibull	*n *≤ 25	25 <*n *	*n *≤ 16	16 <*n *≤ 110	110 <*n *

Therefore, counter intuitively, even for the skewed distributions we tested, it seems like that for a larger sample size (usually more than 25) simply replacing sample mean with the reported median is the best estimate of the sample mean. This is an interesting result and we are not aware that it was previously demonstrated. It gives assurance to meta-analysts that simple replacement of mean with medians in meta-analysis is a viable option. Formula (5), even though taking more parameters into account (the range and the sample size), on average only outperforms the median for small sample sizes. However, a large number of trials used in meta-analyses do have very small number of patients for each arm (as small as 10–15). For these trials, formula (5) seems to give an alternative to just using the median.

When estimating the standard deviation, formula (16) is the best estimate for very small sample sizes (less than 16), after which the range formulas (Range/4 and Range/6) are better. Range/4 formula works best for samples of moderate size (between 16 and about 70), while for really large samples, Range/6 is the best estimator.

Detailed results of each simulation with a skewed distribution are given in the Additional file 2, Additional file 3, Additional file 4, and Additional file 5.

If the reader wants to try these formulas with a different set of data, we have provided an Excel spreadsheet file with the formulas at 

## Effect on the mean difference in meta-analysis

In this section we will discuss the use of these estimating formulas on the effect size for the meta-analysts. When pooling the means from various sources for a meta-analysis, the usual procedure is to calculate differences in the means between the experimental arm of a study and the control arm, *m *_*p *_= *m_c _*- *m_e _*, and the combined variance for each study,  (for example, see [3]). The pooled mean difference is then calculated by using weighted sum of these differences, where the weight is the reciprocal of the combined variance for each study.

To determine whether our estimates make a huge difference when compared to the actual mean difference and variance, we drew two samples of the same size from a same distribution. We applied our methods to the Log-Normal [4, 0.3] distribution since this skewed distribution is frequently encountered in biology and medicine.

**Figure 1:**
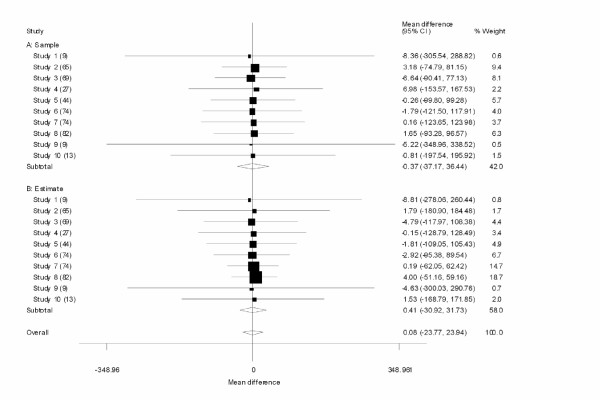
**Meta-Analysis of random data**. After drawing fifteen samples of random sizes (between 8 and 100) from the Log-Normal [4, 0.3] distribution, we used our estimation formulas to estimate the mean and the variance from the median and the range. Then we performed meta-analysis using STATA, treating the real samples as one subgroup and their estimates as another subgroup to determine the results and heterogeneity.

First we ran a test-case meta-analysis. After drawing fifteen samples of random sizes (between 8 and 100) from our distribution, we used our estimation formulas to estimate the mean and the variance from the median and the range. Then we performed meta-analysis using STATA, treating the samples as one subgroup and their estimates as another subgroup to determine the pooled means and heterogeneity. Our results for the weighted mean difference, WMD (see Figure 1) are presented in Table 2.

**Table 2:** Results of our meta-analysis with the real sample data as one subgroup, and our estimates of the sample as the second subgroup.

	**Actual Sample**		**Our Estimate**	
	WMD [95% CI]	% Weight	WMD [95% CI]	% Weight

**Pooled WMD**	-0.37 [-37.17, 36.44]	42.00	0.41 [-30.92, 31.73]	58.00
**Overall pooled WMD**		0.08		100.00
	Heterogeneity statistic	degrees of freedom	P	I-squared
Sample	0.04	9	1.000	0.0%
Estimate	0.04	9	1.000	0.0%
Overall	0.08	19	1.000	0.0%
Overall Test for heterogeneity between sub-groups				
	0.00	1	0.975	
			
Significance test(s) of WMD = 0		Sample	z = 0.02	p = 0.984
		Estimate	z = 0.03	p = 0.980
		Overall	z = 0.01	p = 0.995

^1^I-squared: the variation in WMD attributable to heterogeneity

In order to capture a more consistent measure of the effect of our estimation on pooled mean difference, we repeated this process by varying the number of trials in the meta-analysis from 8 to 100. In particular we are interested in the difference between the real pooled weighted mean difference in the sample group and the pooled weighted mean difference from a meta-analysis using estimated means and variances.

**Figure 2:**
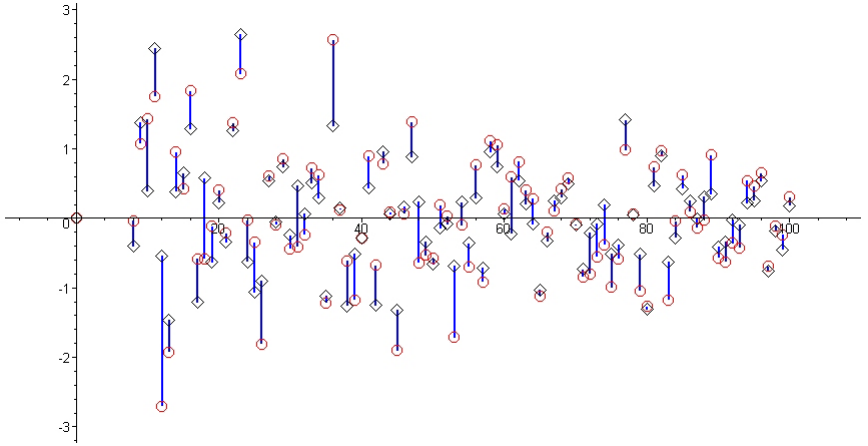
**Actual pooled mean difference and estimated pooled mean difference**. The black diamonds represent the actual pooled mean difference using sample means. The red circles represent the pooled mean differences for the same samples using our estimation formulas (we connected the corresponding symbols for clarity). The horizontal axis represents the number of trials in the meta-analysis (from 8 to 100).

The actual population mean from which we drew samples is 57.11 and the standard deviation is 17.53 (Log-Normal [4, 0.3]). The actual average pooled sample mean difference between two samples (one was control, the other experimental group) was 0.031. Using the medians and range, we estimated the means for each sample, and performed the meta-analysis using these estimates. The average pooled (estimated) mean difference was 0.002, making the difference between the two methods 0.029 (on average). Individually, the pooled means (both, the real sample pooled means, and the estimated pooled means) differed a little more. In Figure 2 the black diamonds represent the actual pooled mean difference using actual sample means. The red circles represent the same pooled mean differences using our estimation formulas (we connected the corresponding symbols for clarity). The horizontal axis represents the number of trials in the meta-analysis (from 8 to 100).

As seen from the Figure 2, the estimates of the mean were fairly accurate and useful. On the other hand, the estimates for the variance were a lot less precise, missing the actual value of the variance by 10 % – 20% (see the Additional Files 1, 2, 3, 4, 5). However, in some situations, using these estimates might still be better than the alternative – excluding the trials which reported the wrong summary data (median instead of mean). Using our estimation method, we can see the effect of such trials on pooled summary measures. In the next section we will illustrate our method in an actual systematic review.

## An illustrative example of the potential value of our methods

American Society of Hematology/ American Society of Oncology (ASH/ASCO) developed practice guidelines for the use of erythropoietin (Epo), a drug whose annuals sales exceed several billions of dollars in the US alone, based on the systematic review of the effects of Epo on various clinical outcomes of interest including improvement of anemia by increase of hemoglobin[4]. The results were expressed as the mean increase in hemoglobin in Epo arm compared with the control. However, a number of the papers reported median increase instead of mean increase and standard deviation. Due to lack of available methods to use median values, the authors of this important review, decided not to use these papers in their meta-analysis. Recently, the Cochrane review was published attempting to provide more updated analysis of the effects of Epo in anemia related to malignancy [5]. The Cochrane reviewers did meta-analyze data to calculate an average weighted mean increase in hemoglobin as the result of Epo treatment. However, the Cochrane investigators could not include the totality of evidence in relation to this outcome since a number of the trials reported data as medians instead of means. Therefore, published meta-analyses related to the effect of Epo in anemia due to malignancy suffer from the phenomena akin to the outcome reporting bias [6] simply due to fact that methods are not yet developed to allow researches to use data medians.

Here we illustrate that it is actually possible to use medians and pool, and improve inclusiveness of meta-analyses. For example, the Cochrane investigators were only able to pool 2 studies [7,8] to evaluate the effect of Epo on change in hemoglobin in the patients with the baseline level of hemoglobin >12 g/dl who underwent chemotherapy. Their results show that on average Epo increases hemoglobin by 2.05 g/dl. However, the Cochrane investigators could not pool data from other available studies in the literature with similar eligibility. ASH/ASCO guidelines listed two other studies that were eligible for the meta-analysis (and two that were not).

**Figure 3:**
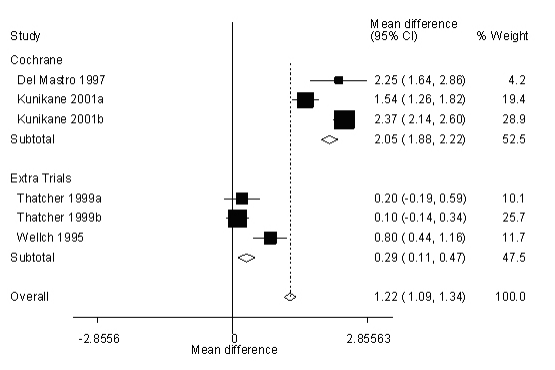
**An example: Meta Analysis with all eligible trials included**. Cochrane investigators [5] were only able to pool two studies to evaluate the effect of Epo on change in hemoglobin in the patients with the baseline level of hemoglobin >12 g/dl who underwent chemotherapy. Their results show that on average Epo increases hemoglobin by 2.05 g/dl. Using our estimation formulas, we were able to include two other studies eligible for this meta-analysis ([9, 10]). The pooled estimate decreased to 1.22 g/dl, i.e., a decrease of approximately 40%.

For the first of these studies [9], by Welch at al, the ASH/ASCO guidelines paper reported the mean hemoglobin change for each of the two arms, the experimental and the control. However, they did not report the data for the standard deviation of these means. Since the size of each arm is 15 patients, our formula (16) provides the best estimate of the standard deviation using the median and the range. We used Figure 1 on page 263 in Welch at al. [9] to estimate the range of the hemoglobin change for each arm and used formula (16) to determine the standard deviation. The ASH/ASCO guidelines paper also reported the difference in medians of hemoglobin response for the largest study eligible for the meta-analysis conducted by Thatcher at al [10]. Thatcher et al do report in their paper ranges of hemoglobin for patients treated by Epo and control. This trial was a three-arm study, in which two doses of Epo were compared against the control. For the purpose of this analysis, we separated the data from each of the Epo arms and compared them against one half of the control group (just like the rest of the studies in the Cochrane review). Using the methods described here, we were able to estimate mean increase (using formula (5)) and standard deviation (using Range/4 formula in both comparisons). When we incorporated these results into the Cochrane meta-analysis, we found that the effect of Epo on mean increase in hemoglobin significantly changed: the pooled estimate decreased from an average of 2.05 g/dl in hemoglobin increase to 1.22 g/dl, i.e., a decrease of approximately 40% (see Figure 3)!

Our estimates come with some uncertainty. To see what effect this uncertainty has on the outcome of our meta-analysis, we varied the estimated means in Thatcher at al by 4% and the estimated standard deviation in both, Thatcher at al and Welch at al, by 10% to 15% (according to sample sizes, as indicated in the Additional Files 1, 2, 3, 4, 5). The summary pooled estimate now ranged from the low of 1.09 to the high of 1.32, which represents a decrease between 36% and 47%.

This example outlines how our method can be potentially useful for meta-analysts. It is important to realize that this example is provided only to illustrate our method. Our goal here is not to challenge the Cochrane review or ASH/ASCO guidelines. Nevertheless, we believe that this example is a good illustration of the potential of our method. While it is common practice that the investigators simply pool what is available to them it is actually not known how often studies are excluded because of reporting a different summary statistic. In future we will attempt to systematically address this issue and evaluate, for example, how often the Cochrane reviews did not pool data from the available median values when they pooled data on continuous outcomes. We hope that availability of our methods to the wider meta-analytic audience may further improve the inclusiveness of all relevant studies for the Cochrane and other meta-analyses.

## Conclusion

We found that a simple formula (5):  can be used to estimate the mean using the values of the median (*m*), low and high end of the range (*a *and *b *, respectively).

Using simulation methods we were able to determine that formula (5) is a best estimator for the mean when dealing with a small sample size. As soon as sample size exceeds 25, the median itself is the best estimator.

The variance can be estimated using the formula (16)



Together with the well-known estimators (Range/4 for a normal distribution, and Range/6 for any random distribution) this formula provides a useful tool for meta-analysts. Using simulations, we determined that for very small samples (up to 15) the best estimator for the variance is the formula (16). When the sample size increases, Range/4 is the best estimator for the standard deviation (and variance) until the sample sizes reach about 70. For large samples (size more than 70) Range/6 is actually the best estimator for the standard deviation (and variance).

In summary, the best estimators for the mean and the standard deviation for different sample sizes are given in Table 3.

**Table 3:** The best estimating formula for an unknown distribution.

Sample Size:	*n *≤ 15	15 <*n *≤ 25	25 <*n *≤ 70	70 <*n *
Estimate Mean	Formula (5)		Median	
Estimate Standard Deviation	Formula (16)	Range/4		Range/6

Using these formulas, we hope to enable meta-analysts use clinical trials even when not all of the information is available and/or reported.

## Competing interests

The author(s) declare that they have no competing interests.

## Authors' contributions

SPH developed most of the formulas for estimation. IH conducted the simulations using Maple. BD conceived of the problem, participated in its framing and coordination, and helped SPH and IH draft the manuscript. All authors read and approved the final manuscript.

## Pre-publication history

The pre-publication history for this paper can be accessed here:



## Supplementary Material

Additional File 1 **Normal distribution. **The top row of the table displays the results of estimating the mean, while the second row displays the results of estimating the standard deviation. Each number in this table represents the average relative error of 200 samples from a Normal distribution. Click here for file

Additional File 2 **Log-Normal distribution. **The top row of the table displays the results of estimating the mean, while the second row displays the results of estimating the standard deviation. Each number in this table represents the average relative error of 200 samples from a Log-Normal distribution. Click here for file

Additional File 3 **Beta Distribution. **The top row of the table displays the results of estimating the mean, while the second row displays the results of estimating the standard deviation. Each number in this table represents the average relative error of 200 samples from a Beta distribution. Click here for file

Additional File 4 **Exponential Distribution. **The top row of the table displays the results of estimating the mean, while the second row displays the results of estimating the standard deviation. Each number in this table represents the average relative error of 200 samples from a Exponential distribution. Click here for file

Additional File 5 **Weibull Distribution. **The top row of the table displays the results of estimating the mean, while the second row displays the results of estimating the standard deviation. Each number in this table represents the average relative error of 200 samples from a Weibull distribution. Click here for file
