# Supplementary material for: Estimating the mean and variance from the median, range, and the size of a sample
Source: BMC Med Res Methodol. 2005 Apr 20;5:13. doi: 10.1186/1471-2288-5-13 (PMC1097734; doi:10.1186/1471-2288-5-13)
Supplement: Additional File 3 — Beta Distribution. The top row of the table displays the results of estimating the mean, while the second row displays the results of estimating the standard deviation. Each number in this table represents the average relative error of 200 samples from a Beta distribution. [file 1471-2288-5-13-S3.pdf]

		The Sample size ranges from 8 to 15.	The Sample size ranges from 16 to 35.	The Sample size ranges from 36 to 72.	The Sample size ranges from 73 to 120 and beyond.
**Approximation of the mean**. Each number in this table represents the average relative error of estimating the sample mean in 200 samples from a Beta distribution.	LEGEND: The median approximation is represented by black crosses, Formula (4) is represented by blue boxes, and Formula (5) by red diamonds.	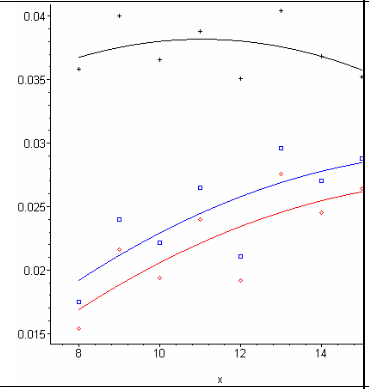	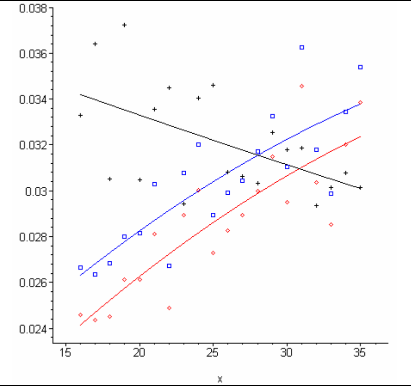	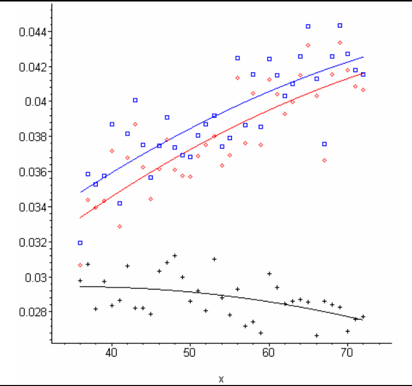	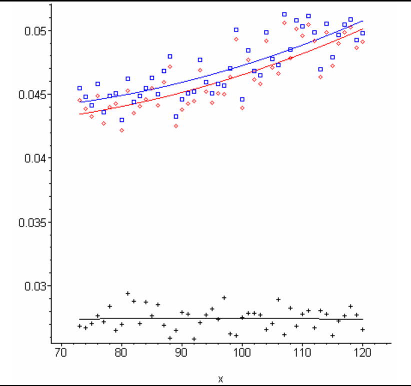
	**Median**	3.73 %	3.21 %	2.88 %	2.74 %
	**Formula (4)**	2.46 %	3.03 %	3.91 %	4.70 %
	**Formula (5)**	2.23 %	2.86 %	3.80 %	4.62 %
	**Conclusion**	Formula (5) is within 2% of the actual sample mean and is performing the best. Formula (4)is almost indistinguishable from Formula (5), and the median is very close behind	All three of these formulas a very close, but the median approximation starts being the best when the sample size reaches about 30.	The median continues to be the best estimator, separating itself from the other two formulas for the sample sizes in this range.	The averages stabilize and remain fairly steady as the sample size increases.
**Approximation of the standard deviation.** Each number in this table represents the average relative error of estimating the sample mean in 200 samples from a Beta distribution.	LEGEND: The Formula (12) is shown using black crosses, Formula (16) is represented by blue boxes; Range/4 by the green circles, and Range/6 by brown diamonds.	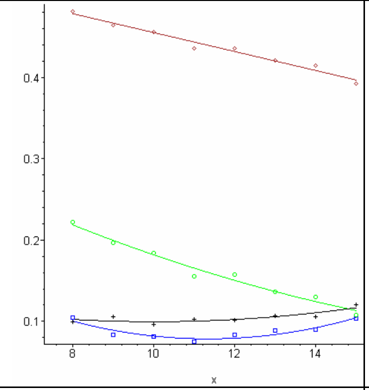	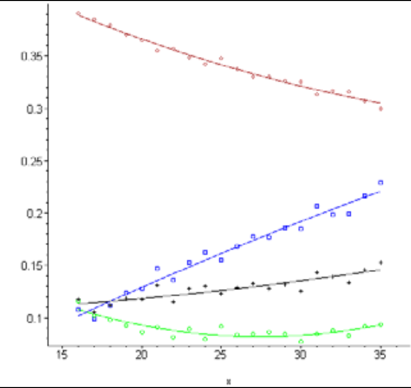	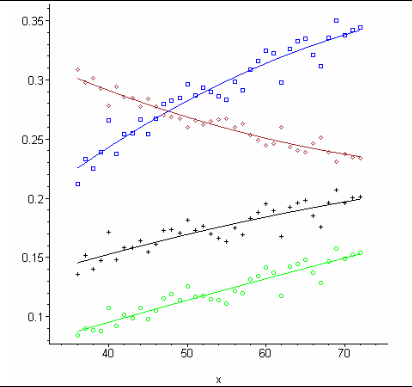	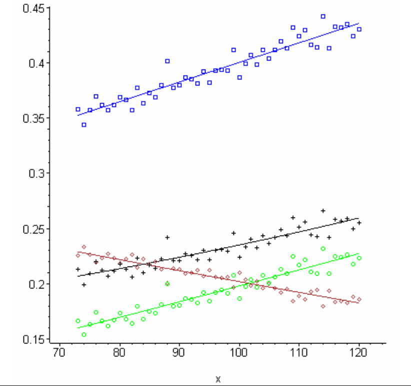
	**Formula (12)**	10.47 %	12.77 %	17.44 %	23.19 %
	**Formula (16)**	8.87 %	16.32 %	29.15 %	39.41 %
	**Range/4**	16.12 %	8.91 %	12.09 %	19.33 %
	**Range/6**	43.75 %	34.21 %	26.34 %	20.56 %
	**Conclusion**	Formula (16) is the best estimator of the standard deviation in this range of sample sizes.	The Range/4 formula takes over as the best estimate for the variance	The Range/4 formula is slowly losing its advantage, and the Range/6 formula is closing in.	The Range/6 takes over the lead in accuracy when the sample size reaches 100, and keeps it as the sample sizes increase.

TABLE 3: Beta Distribution with parameters $a = 9$ and $b = 4$.
